# Scientific Items

**Published:** 1883-08-20

**Authors:** 


### SCIENTIFIC ITEMS.

**The Gradual Cooling of the Earth.**—In a "Treatise on Natural Philosophy," by Professors Sir W. Thompson and P. G. Tait, Sir W. Thompson, speaking of an opinion advanced by Sir Charles Lyell, respecting the possible maintenance of the earth's heat without change throughout countless ages, used words which, says *Knowledge*, may be applied without change of a word to the stupendous theory advanced by Sir C. Siemens not so very long since—such an idea of a practically endless cycle "violates the principles of natural philosophy in exactly the same manner, and to the same degree, as to believe that a clock constructed with a self-winding movement may fulfill the expectations of its ingenious inventor by going forever." The earth is necessarily cooling from century to century; her volcanic energies are certainly diminishing—as certainly, to use an illustration of Sir W. Thomson's, as the quantity of gunpowder in a "monitor" is diminishing when, hour after hour, she is seen to discharge shot and shell, whether at a nearly equable rate or not. without receiving fresh supplies of ammunition.—*Independent Practitioner.*

**Effect of the Castor Oil Plant on Flies.**—The British Medical Journal says that a castor-oil plant was placed accidentally in a room swarming with flies, but almost immediately the flies disappeared, and flies were found under the plant, or clinging to its leaves, dead. The leaves are said to give out a property deadly to insects. Who knows but the mosquito, too, may succumb to castor-oil, and that New Jersey and Staten Island may yet enjoy life, even in the dog-days?—*Microcosm.*

**Octagonal Religious Services in the Eastern Penitentiary, Philadelphia.**—Eigh tclergymen preached simultaneously in the Eastern Penitentiary, Philadelphia, last Sunday, to invisible audiences. This prison is conducted on the principle of solitary confinement. Each prisoner has his own lonely cell. These cells open on eight corridors, radiating from an octagonal centre. The preachers stood at the outer ends of the corridors, and could be heard by the occupants of the cells in their several sections. A group of officials and reporters in the middle of the prison experienced the sensation of listening to eight sermons at once.—*Microcosm.*

**The Coldest Town in the World.**—The coldest inhabited town in the world is, according to L'Union Medicale, not Irkoutsk, as has been formerly believed, but Verchojansk, in Siberia. In this place the mean temperature during the month of January was—43° F., in February, —56° F., in March. —37°. Once the thermometer recorded —81.4° F.

**Tremors of the Earth.**—The London *Times* publishes a synopsis of some papers on the "Tremors of the Earth," by the committee appointed to measure the lunar disturbance of gravity and by Mr. G. Darwin, which contains some statements new to the public. It is considered proved by the men of science engaged that the crust of the earth bends under the weights imposed on it, till, "when the barometer rises an inch over a land area like that of Australia, the increased load of air sinks the entire continent two or three inches below the normal level." The land actually sinks and rises under the pressure of the mass of water thrown upon it by the tides. The maximum of rise and fall on the Atlantic seaboard reaches five inches. The effect is felt at the bottom of the deepest mine, and may reach for an unknown distance. It follows that the crust of the earth must be of exceeding tenacity, uxceeding as a minimum that of granite, and its swaying may be the cause of phenomena hitherto quite unexplained, as, for example, the relation between storm and earthquake. So universal, frequent and unavoidable are these disturbances that the inquiry into the lunar disturbance of gravity has been given up. No depth can be found at which a recording instrument can be placed so as to escape their effeci. The round earth pants, in fact, like a breathing being, under changes always going on above her.—*Journal of Chemistry.*

**Vaccinnating Live Stock.**—M. Pasteur tells the Academy of Sciences of Paris that wonderful results are being obtained in the work of vaccinnating live stock as a preventative of disease. During the last year 80,000 sheep, 4,000 head of cattle and 500 horses have been vaccinated. Before this system was introduced the annual loss from liver-rot in one department was nine per cent., while the loss since then has been reduced over one-half. Among flocks partially vaccinated even the loss is one to ten between the vaccinated and unvaccinated. The experiment was fairly tried, the cattle receiving in care and food the some treatment. Among the 4,562 head of cattle vaccinated during the year there were but eleven deaths, the rate of mortality being reduced from 7.03 per cent. to .24 per cent.—*Jour. Hoalth.*

**An Expert Machinist.**—Charles Sommerville, a machinist employed in the lock works at Stamford, is so expert tn his business that he can cut an ordinary sewing machine needle in two lengthwise, drill a hole through each half, and then fasten them together so accurately that the place where it was separated cannot be seen.—*Hartford Couraut.*

**Large Telephone.**—Among the special features of the Munich electrical exhibition is a giant telephone which transmits music so as to be audible to the entire audience in a large hall.

**Gradual Disappearance of the Spot on the Planet Jupiter.**—The great red spot which has been visible on the surface of the planet Jupiter for several years is reported to be growing fainter, and the early disappearance of this remarkable object seems imminent.

